# Ciliary and non-ciliary expression and function of *PACRG* during vertebrate development

**DOI:** 10.1186/2046-2530-1-13

**Published:** 2012-08-01

**Authors:** Thomas Thumberger, Cathrin Hagenlocher, Matthias Tisler, Tina Beyer, Nina Tietze, Axel Schweickert, Kerstin Feistel, Martin Blum

**Affiliations:** 1 Institute of Zoology, Working group Embryology, University of Hohenheim, Garbenstraße 30, Stuttgart, 70593, Germany; 2 Institute of Zoology, Working group Neural Stem Cells, University of Hohenheim, Garbenstraße 30, Stuttgart, 70593, Germany; 3 Present address: Centre for Organismal Studies (COS), University of Heidelberg, Im Neuenheimer Feld 230, Heidelberg, 69120, Germany

**Keywords:** Cilia, Gastrulation defect, Left-right asymmetry, Leftward flow, Neural tube closure defect, PACRG, Park2, Xenopus

## Abstract

**Background:**

*Park2-co-regulated gene* (*PACRG*) is evolutionarily highly conserved from green algae to mammals. In *Chlamydomonas* and trypanosomes, the PACRG protein associates with flagella. Loss of *PACRG* results in shortened or absent flagella. In mouse the PACRG protein is required for spermatogenesis. The purpose of the present study was to analyze (1) the expression patterns of *PACRG* during vertebrate embryogenesis, and (2) whether the PACRG protein was required for left-right (LR) axis specification through cilia-driven leftward flow in *Xenopus laevis*.

**Methods:**

*PACRG* cDNAs were cloned and expression was analyzed during early embryonic development of *Xenopus*, mouse, rabbit and zebrafish. Antisense morpholino oligonucleotide (MO) mediated gene knockdown was applied in *Xenopus* to investigate LR development at the level of tissue morphology, leftward flow and asymmetric marker gene expression, using timelapse videography, scanning electron microscopy (SEM) and whole-mount *in situ* hybridization. Results were statistically evaluated using Wilcoxon paired and χ^2^ tests.

**Results:**

*PACRG* mRNA expression was found in cells and tissues harboring cilia throughout the vertebrates. Highly localized expression was also detected in the brain. During early development, *PACRG* was specifically localized to epithelia where leftward flow arises, that is, the gastrocoel roof plate (GRP) in *Xenopus*, the posterior notochord (PNC) in mammals and Kupffer’s vesicle (KV) in zebrafish. Besides its association with ciliary axonemes, subcellular localization of PACRG protein was found around the nucleus and in a spotty pattern in the cytoplasm. A green fluorescent protein (GFP) fusion construct preferentially labeled cilia, rendering PACRG a versatile marker for live imaging. Loss-of-function in the frog resulted dose dependently in LR, neural tube closure and gastrulation defects, representing ciliary and non-ciliary functions of PACRG.

**Conclusions:**

The PACRG protein is a novel essential factor of cilia in *Xenopus*.

## Background

*PACRG* was originally identified as a gene related to Parkinson’s disease (PD) in humans [1,2]. In mammals *PACRG* shares a bidirectional promoter with *Park2*, the target gene for early onset juvenile PD. *PACRG* represents an evolutionarily very highly conserved gene, which is present from green algae to mammals [1,3,4]. Although a precise function has yet to be ascribed, the available evidence suggests that the PACRG protein is associated with the ciliary axoneme: antibodies or green fluorescent protein (GFP) fusion proteins detected PACRG in flagellae of *Chlamydomonas reinhardtii*[3] and of *Trypanosoma brucei*[4] as well as in mouse spermatocytes [5]. Parallel RNAi-mediated knockdown of two paralogous genes in trypanosomes resulted in motility-impaired specimens with flagella of apparently normal length but outer microtubule doublet defects [4]. In the viable mutant mouse *quaking* (*qk*^*v*^) male fertility was lost due to a deletion of *PACRG*, which resulted in failure to complete spermatogenesis [5]. Mutant mice were also affected by acquired hydrocephalus due to a defect in ependymal cilia function, resulting in reduced cerebrospinal fluid flow [6]. Structural investigations suggested that PACRG associated with nexin interdoublet links in trypanosomes [4]. In contrast, a localization between A and B tubules was proposed in the axoneme of *Chlamydomonas* flagellae [3]. Non-ciliary localizations were reported as well. PACRG was found in a large molecular chaperone complex containing heat shock proteins 70 and 90 as well as chaperonin components [2]. PACRG was further detected in Lewy bodies: these are neuronal inclusions frequently found in the brain of PD patients that are also positive for Parkin, the protein encoded by *Park2*[2].

Cilia play a pivotal role during early vertebrate embryogenesis [7-10], with the establishment of the LR body axis as the first event where cilia are required [11-14]. During gastrulation a ciliated epithelium forms at the posterior pole of the emerging notochord [15]. This epithelium harbors rotating monocilia, which due to their posterior polarization produce a leftward flow of extracellular fluid. Epithelia vary in shape and size but are structurally and functionally homologous [16]. They comprise the Kupffer’s vesicle (KV) in bony fish, the gastrocoel roof plate (GRP) of amphibian embryos and the PNC in mammals. Experimental or genetic inhibition of flow, ablation or mispolarization of cilia or impairment of ciliary motility in all cases results in LR axis defects [11,13,17,18]. Downstream of leftward flow, the asymmetric *Nodal* gene cascade, consisting of the growth factor *Nodal*, its feedback inhibitor *Lefty* and the homeobox transcription factor *Pitx2*, is initiated in the left lateral plate mesoderm (LPM) and governs asymmetric organ morphogenesis and placement at later stages of development [7].

Here, we asked whether *PACRG* plays a role during embryogenesis as well, specifically during LR axis formation. *PACRG* expression was predominantly found in tissues harboring cilia in early frog, mouse, rabbit and zebrafish embryos. A GFP fusion protein labeled cilia in the frog GRP and epidermis. Gene knockdown in the frog demonstrated an embryonic role of *PACRG* in gastrulation, LR development and neural tube closure.

## Methods

### Cloning of constructs

Total RNA was isolated from embryos of various stages (frog, mouse, rabbit and zebrafish) and cDNAs were prepared using standard protocols. Primers for PCR amplification of *X. laevis PACRG* (accession number JQ771622) were designed based on *X. tropicalis* expressed sequence tags (accession numbers CX959700.1, CU025070.1): untranslated region (UTR) forward 5′-TAGGCAACCGAACGTAAACAACAG-3′; forward 5′-ATGGTGTTTGAGACAAGCAAAGCAACA-3′; reverse 5′-GTTCAGCAAGCAGGATTCAT-3′. In order to clone the enhanced GFP (eGFP) fusion construct, *Bam*HI and *Xho*I restriction sites were introduced into forward and reverse primers, respectively. eGFP was cloned using primers forward 5′-CTCGAGATGGTGAGCAAGGGCGAGGAGC-3′ (including *Xho*I site); reverse 5′-TCTAGATTACTTGTACAGCTCGTCCATG-3′ (including *Xba*I site).

*Xenopus PACRG* and *eGFP* were ligated into the *Bam*HI/*Xba*I linearized CS2+ vector. The *Xenopus* rescue-eGFP construct was cloned using mutated *PACRG* forward primer 5′-ATGGTCTTCGAAACTAGTAAGGCAACA-3′ to prevent morpholino oligonucleotide (MO) binding. Mouse *PACRG* (accession number BC120740.1) was cloned using primers forward 5′-CCCTCTCCTCCCCTAAACTC-3′; reverse 5′-GGTCAGTTCAGCAAGCACG-3′. A rabbit *PACRG* fragment was cloned using primers designed to match regions conserved between human (accession number BC044227.1) and mouse *PACRG* (see above): forward 5′- ATGCCGAAGAGGACTAAACTGCTG-3′; reverse 5′-ACCTACGAGTCTTGCTTGCT-3′ (accession number JQ771623). Zebrafish *PACRG* (accession number ENSDARG00000004736) was cloned using primers forward 5′-ATGAGAACCTTTGAACCTTTGGCTA-3′; reverse 5′-GTTGAGAAGGCAGGACTCGTAGGTGGG-3′.

### RNA *in situ* hybridization, immunohistochemistry and histological analysis

Embryos or explanted larval brains were fixed in MEMFA (1 part of (1M MOPS (pH 7.4, Roth), 20 mM EGTA (Applichem), and 10 mM MgSO_4_,(Applichem)), 8 parts H_2_O and 1 part formaldehyde (37%, Roth)) or 4% paraformaldehyde (PFA, Roth) for 2 h and processed following standard protocols [18]. Digoxigenin-labeled (Roche) RNA probes were prepared from linearized plasmids using SP6 or T7 RNA polymerase (Promega). *In situ* hybridization was according to [19]. Immunohistochemistry was performed on whole-mount embryos fixed in 4% PFA for 1 h at room temperature. Embryos were processed according to standard procedures [18]. Antibodies used include mouse monoclonal antibody directed against acetylated alpha tubulin (1:700; Sigma), rabbit polyclonal antibody directed against PACRG (1:100, Rockland Immunochemicals, Inc.) and Cy2-conjugated or Cy3-conjugated secondary polyclonal rabbit or sheep anti mouse antibodies (Jackson Immunoresearch or Sigma; both 1:250). RNA encoding membrane red fluorescent protein (mRFP) (50 to 100 ng/μl) or rhodamine-B dextran (0.5 to 1.0 μg/μl; Molecular Probes) were used as lineage tracers. For histological analysis embryos were embedded in gelatin-albumin and sectioned on a vibratome at 30 μm (standard) to 40 μm (brain sections). Statistical calculations of marker gene expression patterns were performed using Pearson’s χ^2^ test (statistical R; http://cran.r-project.org/). SEM analysis was performed as described [20]. GRP cilia and cell parameters were determined in a square of 1,000 × 1,000 pixels (magnification 500-fold, corresponding to 86 μm^2^) at the center of GRP in SEM pictures [21]. Cilia lengths, polarization (posterior, central, other) and cell surface areas were determined manually in ImageJ [22]. Ciliation rates were calculated as the ratio of cilia over cells (separately in each GRP SEM photograph). Posterior polarization was quantified for each GRP and statistical significances were calculated by Student’s t test in statistical R (http://cran.r-project.org/). The whiskers of the box plots extend to maximal 1.5 × interquartile range (IQR), outliers are displayed as dots.

### Microinjections and MO-mediated knockdown of frog *PACRG*

Embryos were injected at the four to eight cell stage using a Harvard Apparatus set-up. Drop size was calibrated to about 7–8 nl/injection. Morpholinos (Gene Tools, Philomath, OR, USA) were used at 0.4-2 pmol/embryo as indicated. Lineage tracer RNAs were prepared using the Ambion message machine kit (Ambion) and diluted to a concentration of about 50–100 ng/μl. In all experiments care was taken to exclusively use four to eight cell embryos with a clear dorsoventral segregation of pigment [23,24], and only correctly targeted specimens (controlled by coinjected lineage tracer) were processed for further analysis. The AUG blocking MO for frog *PACRG* (*PACRG*-MO) comprised 5′-TGCTTGTCTCAAACACCATATTCAC-3′.

### Video analysis of cilia, blastopore closure and leftward flow

Fluorescent *in vivo* imaging of epidermal and GRP cilia was performed following injection of 80 ng/μl *PACRG-eGFP* mRNA. Timelapse sequences were recorded on a Zeiss Axioskop equipped with a CCD camera (AxioCam Hsm, Zeiss) using AxioVision 4.6 (Zeiss) at 62 fps (beating cilia) or 2 fps (leftward flow). For blastopore closure timelapse acquisition, specimens were mounted onto an inverse microscope in a glass-bottom Petri dish onto a nitex mesh and cultivated in 0.1 × MBSH (Modified Barth‘s Saline). Timelapse movies were acquired at one frame every 2 minutes. Preparation of dorsal explants, recording of timelapse movies, processing and analysis of leftward flow were according to [18]. Significances were calculated by Student’s t test in statistical R (http://cran.r-project.org/). The whiskers of the box plot extend to maximal 1.5 × IQR.

## Results and discussion

### Cloning and expression analysis of *PACRG* mRNA during vertebrate embryonic development

**Figure 1:**
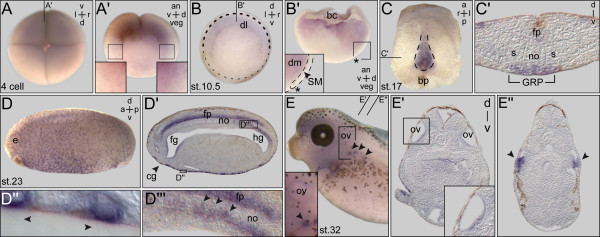
***PACRG* expression during early* Xenopus* development. (A)** Expression at the four-cell stage (top view). **(A’)** Sagittal hemisection of embryo shown in (A). Enlargements (boxes) indicate higher expression levels on the dorsal side. **(B)** Gastrula embryo. Persistent differences in staining intensities between dorsal and ventral side. **(B’)** Sagittal hemisection (plane indicated in (B)) revealing mRNA localization in deep mesoderm. Note that the superficial mesoderm (SM) was free of *PACRG* mRNA (inset in (B’)). Dorsal lip marked by asterisk. **(C)** Expression in the gastrocoel roof plate (GRP) at stage 17 (dorsal explant shown in ventral view). **(C’)** Histological section (plane marked in (C)). **(D)** Expression at stage 23 in ciliated cells of the epidermis and the floor plate (sagittal section shown in **(D’)**). Enlargements show multiciliated skin cells (**(D”)**; cilia indicated by arrowheads) and GRP cells after intercalation into the notochord (**(D”’)**; arrowheads). **(E)** Staining in the otic vesicle and nephrostomes (arrowheads). **(E’,E”)** Histological sections (levels indicated in (E)) highlighting expression in the otic vesicle (E’) and nephrostomes ((E”), arrowheads). a = anterior; an = animal; bc = blastocoel; bp = blastopore; d = dorsal; dl = dorsal lip; dm = deep mesoderm; fg = foregut; fp = floor plate; hg = hindgut; l = left; no = notochord; ov = otic vesicle; p = posterior; r = right; s = somite; v = ventral; veg = vegetal.

*PACRG* cDNAs were cloned from frog, mouse, rabbit and zebrafish. Alignment of deduced amino acid sequences revealed high conservation between vertebrate species (Additional file 1: Figure S1). Expression patterns during development have so far not been described in vertebrates with the exception of zebrafish, where data from an *in situ* screen have been deposited into the Zfin database [25]. Embryonic expression was analyzed by whole mount *in situ* hybridization (WMISH) of staged embryos. In *Xenopus*, maternal mRNA was detected at the four-cell stage (Figure 1A). A punctate pattern was observed, which appeared slightly enriched on the dorsal compared to the ventral side, and more pronounced animally compared to the vegetal half (Figure 1A’). Zygotic expression was detected at the onset of gastrulation in the marginal zone, with again stronger signals on the dorsal side (Figure 1B). A hemisection revealed expression in the deep mesodermal layer (Figure 1B’). As cilia have not been reported during these early stages, expression indicated potential non-ciliary functions of *PACRG*. The first cilia-related staining was found in the GRP from stage 13 onwards (Figure 1C,C’ and data not shown). No signals were seen in the superficial mesoderm (SM), from which the GRP derives during gastrulation (Figure 1B’). Onset of *PACRG* expression thus correlated with the outgrowth of cilia, unlike other ciliary genes such as *Foxj1* or *dnah9*, which are already induced in the SM and persist to be expressed in the GRP proper [18,26]. Multiciliated epidermal cells started to show *PACRG* mRNA localization at stage 17 (Figure 1C’), and maintained expression throughout embryogenesis (Figure 1D,E). Additional prominent sites of expression in ciliated cells were seen in the floor plate (Figure 1D’), nephrostomes (Figure 1E,E”) and otic vesicle of the 2-day tadpole (Figure 1E,E’). Interestingly, notochordal GRP cells maintained *PACRG* staining at post-flow stages, when cells intercalated into the overlying notochord (Figure 1D”’; [27]).

**Figure 2:**
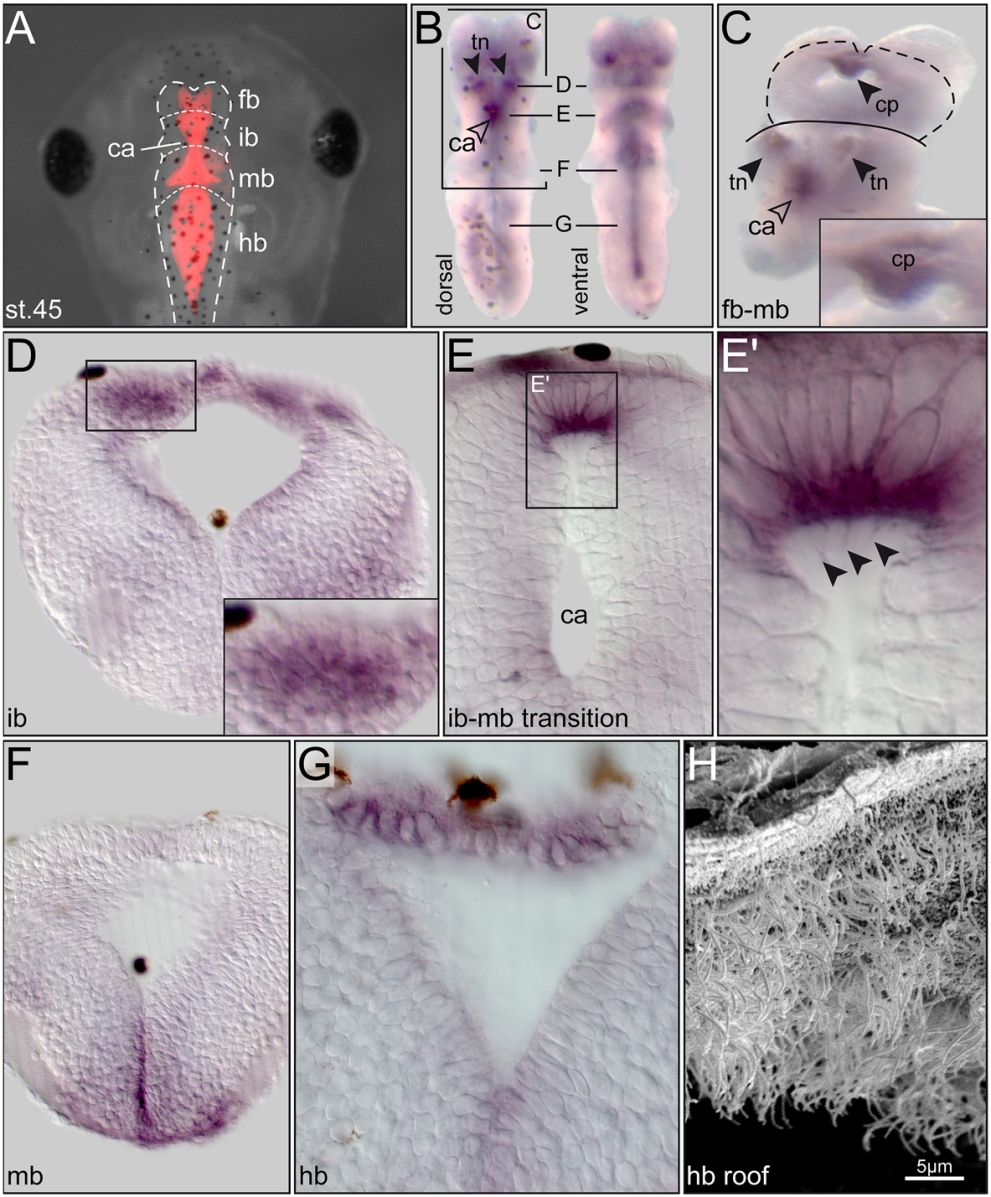
***PACRG* expression in the developing* Xenopus* brain. (A)** Brain anatomy of the 3-day tadpole (stage 45), as highlighted by rhodamine dextran injection into the hindbrain ventricle. **(B)** Isolated brain following *in situ* hybridization with an antisense *PACRG* probe, shown in dorsal (left) and ventral (right) view. **(C)** Whole-mount of brain in which the hindbrain was removed and a dorsal cut was introduced between forebrain and interbrain to expose the choroid plexus (higher magnification shown in inset). **(D-G)** Transversal histological sections; levels indicated in (B), dorsal side up. (D) Staining in the thalamic nuclei. (E) Localized expression in roof of cerebral aqueduct (cilia highlighted by arrowheads in the enlargement shown in **(E’)**). (F) Expression in ventral midline of midbrain region. (G) Staining in roof of hindbrain. **(H)** Scanning electron micrograph, demonstrating multiciliated cells in roof of hindbrain. ca = cerebral aqueduct; cp = choroid plexus; fb = forebrain; hb = hindbrain; ib = interbrain; mb = midbrain; tn = thalamic nucleus. Note that dark brown spots in whole-mount brain specimens (B) and sections (D-G) represent melanocytes.

Expression in the central nervous system was investigated in *Xenopus* whole-mount brain explants. The brain was interesting, because (1) adult *qk*^*v/*^*qk*^*v*^ mice develop hydrocephalus, a phenotype related to impaired cilia function [6], and (2) *PACRG* mRNA and protein was described to be expressed in regional brain areas of newborn and adult mice such as the lateral ventricles, the third and fourth ventricle, the aqueduct of Sylvius and the choroid plexus [28,29]. Brain samples were analyzed in 3-day (stage 40, data not shown) and 5-day (stage 45) tadpoles with comparable results (Figure 2). *PACRG*-specific signals were detected in multiciliated choroid plexus cells [28], thalamic nuclei and in the ventral midline (Figure 2A-C). Whether *PACRG,* in the cells of the ventral midline, has a ciliary or non-ciliary expression has not been determined so far. Interestingly, the specific expression in the thalamic nuclei (Figure 2D) correlates with a major site of non-dopaminergic neuron degeneration in the brain of PD patients [30]. Histological sections revealed staining in ciliated cells at the dorsal roof of the cerebral aqueduct (that is, the forming aqueduct of Sylvius; Figure 2E,E’) and in the roof of the hindbrain (Figure 2G,H). In summary, expression patterns homologous to the one in the adult mouse brain were already visible during frog tadpole development, suggesting a function in the developing embryonic brain.

**Figure 3:**
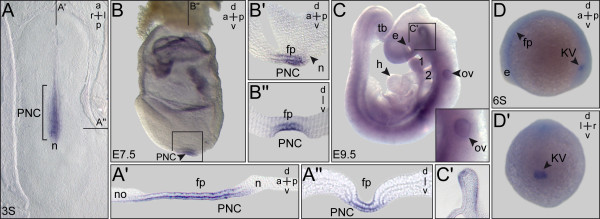
***PACRG* expression during early rabbit, mouse and zebrafish development.** Whole-mount *in situ* hybridization of staged embryos. **(A)** Rabbit. Three somite (3S) stage blastodisc revealing *PACRG* expression in the posterior notochord (PNC) and floor plate. **(A’)** Sagittal section. **(A”)** Transversal section (level marked in (A)). Note that the sagittal section revealed absence of *PACRG* from the node (A’). **(B,C)** Mouse. (B) E7.5 late headfold mouse embryo displaying *PACRG* expression in the PNC and floor plate. **(B’)** Sagittal section. **(B”)** Transversal histological section. **(C)** E9.5 embryo. Otic vesicle shown in higher magnification in inset. Box indicates brain region of which a histological section is provided in **(C’)**. (C’) Histological sagittal section, revealing staining in ventral midline of the brain. **(D)** 6-Somite stage zebrafish embryo. *PACRG* expression in the Kupffer’s vesicle (KV) and floor plate. a = anterior; d = dorsal; e = eye; fp = floor plate; h = heart; l = left; n = node; no = notochord; ov = otic vesicle; p = posterior; r = right; s = somite; tb = tail bud; v = ventral.

Rabbit, mouse and zebrafish embryos were investigated with a specific emphasis on the ciliated epithelia relevant for leftward flow. Figure 3 shows that *PACRG* mRNA was present in the PNC of rabbit and mouse as well as in the zebrafish KV. mRNA transcripts were localized to other ciliated tissues as well, such as the floor plate in all species and in the mouse otic vesicle. Comparable to *Xenopus* signals were also detected in the ventral midline of the developing mouse brain at E9.5 (Figure 3C,C’). These data indicate that primary amino acid sequences and embryonic expression patterns were likewise conserved among the vertebrates. This notion is further supported by data available from the above-mentioned zebrafish expression screen. There, *PACRG* expression was additionally annotated in the otic vesicle, midbrain, pronephros, tegmentum and lateral line organ, all of which are ciliated structures [25]. Taken together, our expression analysis demonstrated a highly conserved pattern of *PACRG* mRNA localization in ciliated embryonic tissues, consistent with the axonemal localization reported in *Chlamydomonas* and trypanosomes [3,4]. In addition, signals were found in non-ciliated cells during early cleavage stages and in brain regions not analyzed for the presence of cilia as yet.

### PACRG protein localizes to cilia and intracellular compartments

**Figure 4:**
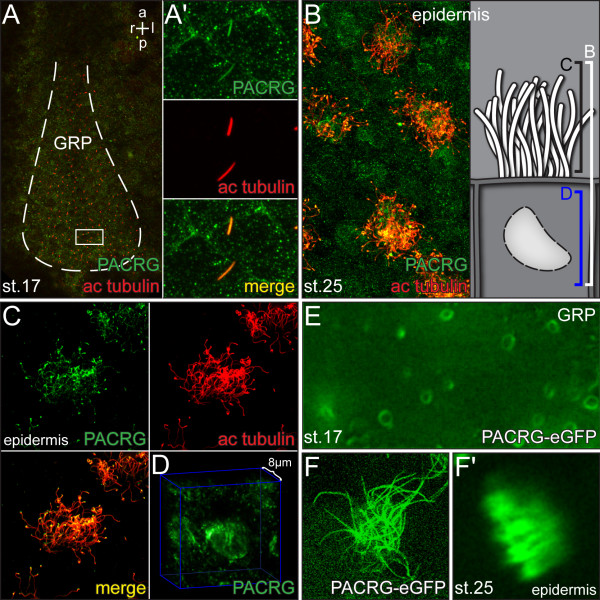
**PACRG protein localization. (A-D)** Immunohistochemistry. PACRG shown in green and acetylated tubulin in red. (A) Ventral view of gastrocoel roof plate (GRP) (dashed white line) at stage 17. **(A’)** Enlargement of GRP cilia. (B) Epidermis. Overview and schematic depiction of multiciliated epidermal cell. (C,D) Enlargements of cilia (C) and cytoplasmic PACRG staining (D). Note that within the cell PACRG localizes to vesicle-like structures and the perinuclear region. **(E,F)** Expression of a PACRG-enhanced green fluorescent protein (PACRG-eGFP) fusion protein. (E) Maximum intensity projection of timelapse movie of stage 17 GRP cilia, demonstrating elliptic shape of ciliary beating. **(F)** Squash preparation and **(F’)** maximum intensity projection of timelapse movie of stage 25 multiciliated skin cell. a = anterior; ac = acetylated; l = left; p = posterior; r = right.

Further analyses of *PACRG* were performed in *Xenopus* embryos, as this model organism is ideally suited to study LR asymmetry [31]. To confirm axonemal protein localization, immunohistochemistry was applied in which axonemal microtubules and PACRG were stained simultaneously. Figure 4A demonstrates PACRG localization along monocilia of GRP cells at stage 17. Additional PACRG signals were detected in a punctate pattern in the cytoplasm and at the plasma membrane (Figure 4A’), indicating axonemal as well as cytoplasmic functions. Localization to multiciliated skin cells of stage 25 tadpoles is depicted in Figure 4B. Interestingly, PACRG was again found within the cell as well. Besides scattered punctae a concentration was seen around the nucleus (Figure 4D), which was not further characterized. PACRG localization to cytoplasmic vesicles and to the perinuclear area have previously been reported in cultured primary neurons from the mouse midbrain [2], supporting the specificity of the observed patterns. As cilia in the GRP, nephrostomes, otic vesicle, brain and on epidermal cells are all motile, we wondered whether PACRG localized to primary immotile cilia as well. Primary cilia have been described in the pronephric duct of *Xenopus* tadpoles [32]. We therefore analyzed PACRG expression by immunohistochemistry at stage 37/38. As shown in Additional file 2: Figure S2, a faint though clear signal was detected along the axoneme of primary cilia in the pronephric duct. In summary, PACRG was found to localize to both motile and immotile cilia.

In order to confirm the immunohistochemistry data, we injected a *PACRG-eGFP* fusion construct into four-cell *Xenopus* embryos and targeted the mRNA to the GRP or epidermal cells. As shown in Figure 4E,F, the fusion protein marked both monocilia on the GRP and cilia of multiciliated skin cells. Vesicle-like structures and the perinuclear region were positive for the fusion protein as well (not shown), demonstrating that the fusion protein localized in an identical manner as the endogenous PACRG.

The ciliary localization of PACRG-eGFP afforded the opportunity of testing whether this fusion protein enabled live imaging of motile cilia in the frog *Xenopus*. The thickness of dorsal explants and the high yolk content of cells resulted in scattering of polarized light, which prevented *in vivo* imaging of GRP cilia in top view in the past [13]. Additional file 3, movie 1 shows a field of GRP cilia, confirming their rotational and, due to the posterior tilt, elliptical beat pattern (see also Figure 4E). The whip-like wave form of epidermal cilia bundles could likewise be recorded (Additional file 3:  Movie 1). These data demonstrated that PACRG-eGFP could be used as a cilia marker for live imaging in frog, and perhaps in other vertebrate model organisms as well.

### LR axis defects in *PACRG* morphants

In order to investigate the function of *PACRG* during *Xenopus* LR development, an antisense morpholino oligonucleotide (MO) was designed which targeted the translational start site. *PACRG-*MO or a random control MO (co-MO) were injected into the GRP lineage by targeting the dorsal marginal zone at the four-cell stage as described [18]. Embryos were cultured until control uninjected specimens reached stage 34. Dose-dependently a series of axial defects were recorded (see below). Following injections of 0.4 pmol *PACRG-*MO per embryo specimens developed with wild-type dorsoanterior index (DAI; [33]) of 5 (n = 89/91). Alterations of dorsoanterior development (DAI ≠ 5) frequently indicate midline defects, which inevitably cause altered LR marker gene expression and organ situs [34,35]. Therefore LR parameters were only evaluated in DAI5 *PACRG* morphants.

**Figure 5:**
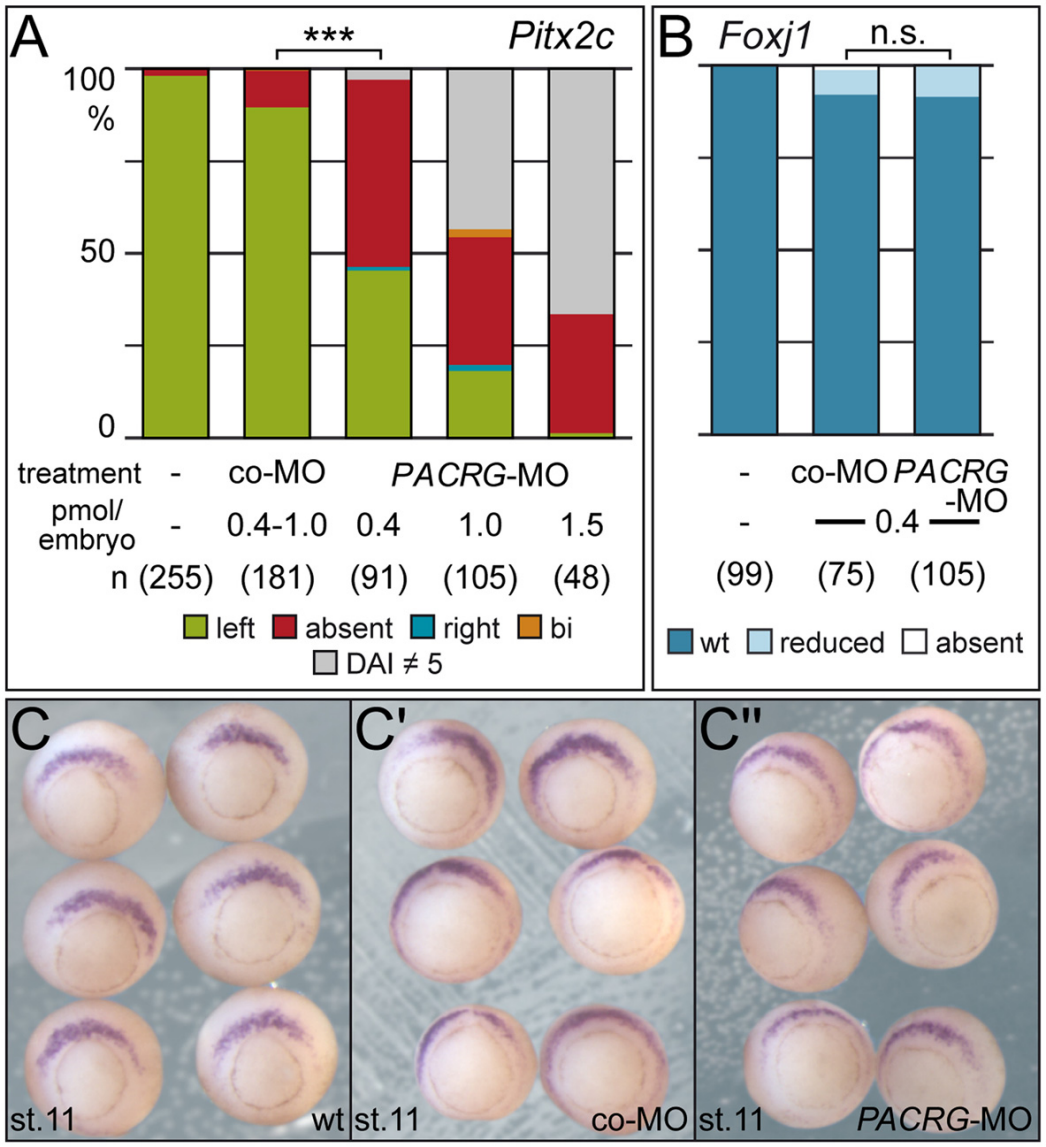
**Impaired laterality in* PACRG* morphants in the absence of superficial mesoderm (SM) defects. (A)***Pitx2c* gene expression in the lateral plate mesoderm (LPM). Note that the morpholino oligonucleotide (MO) dose chosen (0.4 pmol/embryo) did not affect dorsoanterior development. **(B,C)** Unaltered *Foxj1* expression levels in the SM of morphants. Assessment of expression levels was as described previously [36]. Embryos in (C) are shown in vegetal view, dorsal side up. Numbers in brackets represent number of analyzed specimens. *** = very highly significant (*P* <0.001); n.s., not significant (*P* = 0.891).

In a first set of experiments we asked whether *PACRG* was required for LR development. To assess the induction of the asymmetric Nodal cascade in the left LPM expression of *Pitx2c* in 2-day tadpoles was analyzed following *PACRG* knockdown. As shown in Figure 5A*Pitx2c* was predominantly absent in the left LPM of morphant specimens in a dose-dependent manner. Next we wondered whether the SM was specified normally, as SM defects result in aberrant laterality as well [21,31]. To that end expression of *Foxj1*, the master control gene of motile cilia [26] was assessed at stage 11. Figure 5B-C” demonstrates that *Foxj1* was not affected by knockdown of *PACRG*, indicating that the SM was specified correctly and *PACRG* should be required during flow stages.

**Figure 6:**
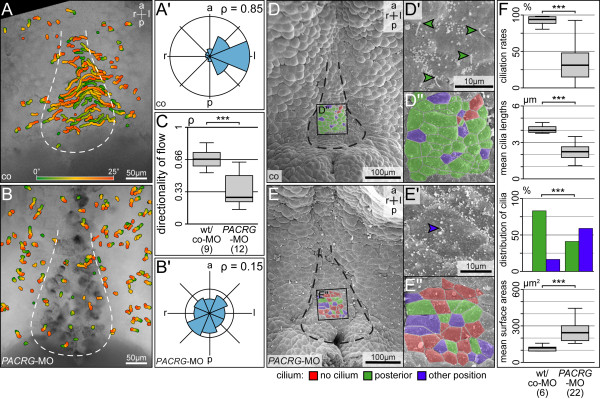
**Absence of leftward flow and altered gastrocoel roof plate (GRP) morphology in *PACRG *morphants. (A,B)** Loss of leftward flow in *PACRG* morphant GRPs. Gradient time trail (GTT) projection of directed bead trajectories (25 s, indicated in color bar). **(A’,B’)** Circular histograms of mean angles of trajectories. **(C)** Quality of flow, as depicted by the dimensionless number rho. **(D,E)** SEM analysis of wild-type (D) and *PACRG* morphant (E) dorsal explants. GRP area indicated by dashed line. Note the loss of posterior polarization of cilia in enlargements of characteristic cells in **(D’,E’)**, and the marked increase in non-ciliated cells **(D”,E”)**. **(F)** Altered ciliation rates, mean cilia lengths, polarization of residual cilia and mean apical cell surface areas, as assessed in standardized central GRP areas of 86 μm^2^ each [21].

To analyze leftward flow, dorsal explants of co-MO and *PACRG*-MO injected embryos were prepared, fluorescent beads were added and timelapse videos were recorded and evaluated as described [17,18]. Additional file 4: Movie 2 and Figure 6A-C show that very few directed beads were detectable in *PACRG* morphants, and quality of flow was severely affected (*P* <0.001). As a measure of flow quality, the dimensionless number rho was used, which represents the mean resultant length of all bead trajectory angles [18], with a rho value of 1 representing beads moving in one direction and rho = 0 representing randomness of bead trajectories. As impaired flow frequently correlates with altered GRP morphology, ciliation and/or cilia function [17,18,21], dorsal explants were examined by SEM. The outline of the GRP was deformed in morphants as compared to wild-type specimens (Figure 6D,E). The mean ciliation rate dropped significantly, and mean cilia lengths and rate of posterior polarization of residual cilia were reduced as well (Figure 6F). In addition the mean apical surface area of morphant GRP cells was enlarged (Figure 6F). Taken together, these results demonstrate that *PACRG* was required for GRP morphology, ciliation, leftward flow and thus left-asymmetric initiation of the nodal signaling cascade in the frog *Xenopus*. The lack of a like phenotype in mouse, where LR defects were not recorded in the *qk*^*v*^ mutant which harbors a *PACRG* deletion, might be due to functional redundancy, as a PACRG-related protein has been annotated in the database (NM_025755.3; [37]). A second *PACRG* gene is not a unique feature of mammals, as trypanosomes have two genes as well [4]. *PARK2 co-regulated-like* has been annotated in *Xenopus* as well (http://www.xenbase.org), however, expression and function have not been assessed so far. Sequences differ such that our MO would not have targeted translation of *PACRG-l*. It remains to be seen if and to what extend *PACRG* and *PACRG-l* complement each other.

### Gastrulation and neural tube closure defects in morphants

**Figure 7:**
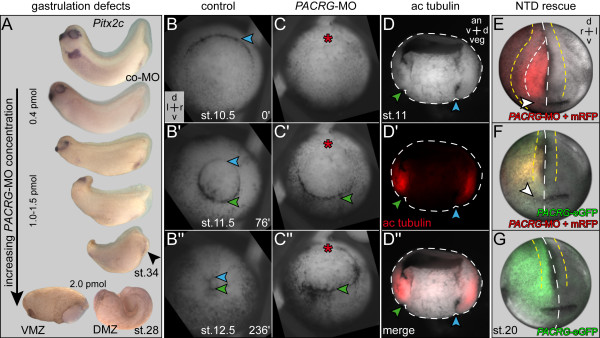
**Gastrulation and neural tube closure defects in high dose* PACRG *morphant embryos. (A)** Phenotypes. Note that with increasing morpholino oligonucleotide (MO) doses the anterior-posterior axis shortened, head structures were lost and the blastopore (arrowhead) failed to close. At 2 pmol/embryo (bottom) gastrulation was arrested on the injected side. **(B,C)** Still frames taken from timelapse movies (Additional file 5: Movie 3) at the stages indicated. (B) Uninjected control specimen. (C) Embryo injected with 2 pmol of *PACRG-*MO into the dorsal marginal zone at the four cell stage. Note that the dorsal lip did not form in morphant, while the uninjected ventral side gastrulated normally and in time. **(D)** Immunohistochemistry with an antibody against acetylated tubulin (red), demonstrating non-dynamic microtubules in the deep mesoderm of the marginal zone of the stage 11 gastrula embryo. **(E,F)** Rescue of neural tube closure defect (NTD) in morphants by parallel injection of a mutated *PACRG*-eGFP construct such that MO binding was abolished (rescue construct; see also Methods). (E) Unilateral right-sided MO injection (0.5 pmol) into the dorsal marginal zone resulted in NTD and loss of cement gland (white arrowhead) on the injected side. (F) Rescue of NTD and partial rescue of the cement gland. **(G)** Wild-type development upon overexpression of *PACRG*-eGFP mRNA. Yellow dashed line = lateral border of neural folds; white dashed line = dorsal midline; blue arrowheads = dorsal lip; green arrowheads = ventral lip; red asterisk = lack of dorsal lip formation. a = anterior; an = animal; d = dorsal; l = left; r = right; veg = vegetal.

At MO concentrations >0.4 pmol/embryo a series of phenotypes was encountered, ranging from mild neural tube closure defects to microcephaly or complete loss of cranial structures (Figure 7A). At doses ≥2 pmol/embryo, severe gastrulation defects occurred, with a failure of blastopore closure on the injected side. In order to follow the appearance of high dose phenotypes during development, timelapse movies were recorded. Additional file 5: Movie 3 and single frames taken from the onset of dorsal lip formation through blastopore closure demonstrate that the dorsal lip did not form in morphants as compared to control embryos (Figure 7B,C). Notably, the ventral lip, which was not targeted by the MO, appeared in time (Figure 7C’). Dorsal lip formation requires apical constriction in order to give rise to bottle cells [38]. Interestingly, apical constriction of bottle cells was shown to be dependent on stable microtubules [38,39]. PACRG binds to and bundles microtubules *in vitro*[40], suggesting that PACRG was required for microtubule function in bottle cell apical constriction. The expression pattern of acetylated alpha tubulin in the deep mesoderm of the marginal zone (reflecting stable microtubules), which colocalizes to *PACRG* mRNA (see also Figure 1B’, and Figure 7D’), supports this notion.

Neural tube closure depends on apical constriction of neural plate cells as well, and as with bottle cell formation this process has been shown to require intact microtubules [41]. Neural tube closure defects (NTD) seen at intermediate *PACRG*-MO doses might therefore be related to altered assembly of parallel microtubule arrays as well. NTD represented a specific *PACRG* phenotype, as we were able to rescue closure by coinjection of *PACRG-eGFP* mRNA using a construct in which the MO binding site was mutated (Figure 7E-G). In summary, intermediate and high doses of MO caused phenotypes unrelated to cilia but associated with intracellular arrangement of ordered microtubule bundles.

## Conclusions

Our study of *PACRG* in four vertebrate model organisms revealed a pronounced degree of conservation at the level of amino acid sequences and embryonic expression. In particular, *PACRG* was highly correlated with motile cilia during development, an aspect that we confirmed in depth by our functional analysis of LR development in *PACRG* morphants in *Xenopus*. The remarkable non-ciliary *PACRG* functions are worth being analyzed in greater depth in future studies. The preliminary evidence presented here points to a more general role related to non-dynamic microtubules in their recently shown involvement in apical cell constriction. In addition, the expression in the embryonic brain at sites where lesions in populations of non-dopaminergic neurons occur in PD patients may deserve further attention. Lastly, *PACRG* may serve as a versatile marker of motile cilia in live imaging.

## Competing interests

The authors declare that they have no competing interests.

## Authors’ contributions

TT carried out live imaging, *PACRG* mRNA and protein expression analysis in *Xenopus* and zebrafish, most of the functional experiments, prepared the figures and movies and helped to draft the manuscript; CH performed the expression and SEM analyses in the brain; MT participated in the morphant analysis; TB conducted the SEM analysis; NT cloned and analyzed *PACRG* expression in rabbit and mouse; KF analyzed the brain expression patterns and ventricular ciliation; AS contributed to the interpretation of the experimental data; MB supervised the study, interpreted the data and wrote the manuscript. All authors read and approved the final manuscript.

## Supplementary Material

Additional file 1 **Additional Figure 1 High conservation of PACRG amino acid sequences from zebrafish, human, rabbit, mouse and *Xenopus laevis*.** Alignment of amino acid sequences derived from zebrafish (ENSDARG00000004736), human (BC044227.1), rabbit (JQ771623) mouse (BC120740.1) and *Xenopus laevis* (JQ771622) cDNAs. Variations were restricted to the N-terminal part, encoded mostly in exon 1. Note that no protein domains of known function have as yet been ascribed to PACRG. Amino acids derived from rabbit primers used for PCR amplification are indicated with blue rectangles (see also Methods). Click here for file

Additional file 2 **Additional Figure 2 Localization of PACRG to primary cilia of the pronephric duct.** Tadpoles at stage 37/38 were fixed and processed for immunohistochemistry with antibodies specific for PACRG (green) and acetylated tubulin (red). Specimens were sectioned on a vibratome (30μm) and viewed in the confocal laser scanning microscope. The lumen of the pronephric duct is outlined by a white dashed line. White rectangles indicate regions shown in higher magnification in the lower left corner. Click here for file

Additional file 3 **Movie 1 A *PACRG*-eGFP fusion construct labels monociliated and multiciliated cells*in vivo*.** GRP cilia (stage 17; left) and multiciliated skin cells (stage 25; right) were labeled by injection of *PACRG*-eGFP at the four-cell stage. The first frame shows maximum intensity projection of timelapse movies, which play in real time. Note that cilia were imaged in top-down view, which in *Xenopus* due to the high yolk content of cells is not possible without labeling of cilia. Click here for file

Additional file 4 **Movie 2 Absence of leftward flow in* PACRG* morphants.** Timelapse sequences of dorsal explant cultures to which fluorescent beads were added. Specimens were mounted dorsal side down (anterior to the top) and are viewed from the ventral side. Movie runs at 40 × real time. Opening frame indicates orientation of GRP (white dashed lines). Videos were processed to yield gradient time trails (GTTs), that is, color-coded tracks of beads which reveal direction of transport and velocity of particles (from green to red; 25 s; [18]). Note that robust leftward flow of control specimen (left) was absent in morphant (right). Click here for file

Additional file 5 **Movie 3 Gastrulation defects in *PACRG *morphants.** Timelapse sequences of control (left) and *PACRG* morphant embryo (right) from stage 9–12.5. *PACRG*-MO was injected into the prospective dorsal marginal zone at the 4-cell stage. Movies were recorded at 0.5 frames per minute and pause at stages 10 and 11 to demonstrate absence (red asterisk) of dorsal lip (blue arrowhead) formation and gastrulation via the ventral lip (green arrowhead) in morphant sample. Note that the unmanipulated ventral lip formed at the same time as in the uninjected control embryo. Embryos are shown in vegetal view, dorsal side up. Click here for file
