# Supplementary figures and images for: Ciliary and non-ciliary expression and function of PACRG during vertebrate development
Source: Cilia. 2012 Aug 1;1:13. doi: 10.1186/2046-2530-1-13 (PMC3555705; doi:10.1186/2046-2530-1-13)

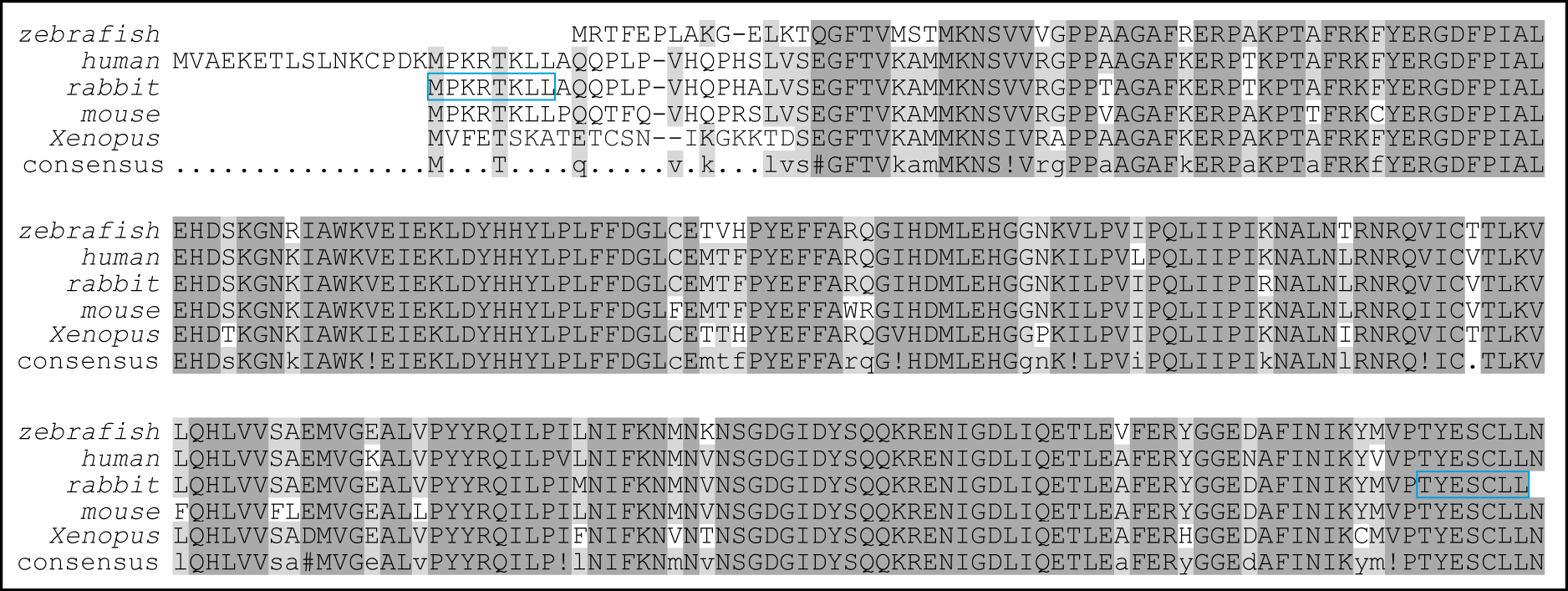

Supplement: Additional file 1 — Additional Figure 1 High conservation of PACRG amino acid sequences from zebrafish, human, rabbit, mouse and Xenopus laevis. Alignment of amino acid sequences derived from zebrafish (ENSDARG00000004736), human (BC044227.1), rabbit (JQ771623) mouse (BC120740.1) and Xenopus laevis (JQ771622) cDNAs. Variations were restricted to the N-terminal part, encoded mostly in exon 1. Note that no protein domains of known function have as yet been ascribed to PACRG. Amino acids derived from rabbit primers used for PCR amplification are indicated with blue rectangles (see also Methods). [file 2046-2530-1-13-S1.jpeg]

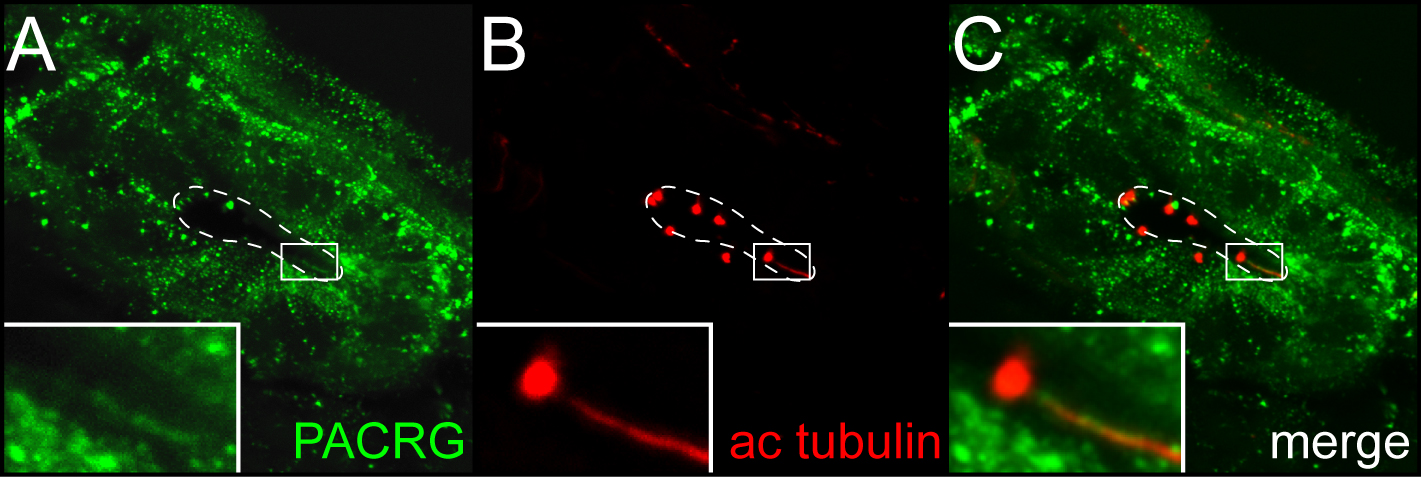

Supplement: Additional file 2 — Additional Figure 2 Localization of PACRG to primary cilia of the pronephric duct. Tadpoles at stage 37/38 were fixed and processed for immunohistochemistry with antibodies specific for PACRG (green) and acetylated tubulin (red). Specimens were sectioned on a vibratome (30μm) and viewed in the confocal laser scanning microscope. The lumen of the pronephric duct is outlined by a white dashed line. White rectangles indicate regions shown in higher magnification in the lower left corner. [file 2046-2530-1-13-S2.jpeg]
